# What Is Stochastic Resonance? Definitions, Misconceptions, Debates, and Its Relevance to Biology

**DOI:** 10.1371/journal.pcbi.1000348

**Published:** 2009-05-29

**Authors:** Mark D. McDonnell, Derek Abbott

**Affiliations:** 1 Institute for Telecommunications Research, University of South Australia, Mawson Lakes, South Australia, Australia; 2 Centre for Biomedical Engineering and School of Electrical & Electronic Engineering, University of Adelaide, Adelaide, South Australia, Australia; University College London, United Kingdom

## Abstract

Stochastic resonance is said to be observed when increases in levels of unpredictable fluctuations—e.g., random noise—cause an increase in a metric of the quality of signal transmission or detection performance, rather than a decrease. This counterintuitive effect relies on system nonlinearities and on some parameter ranges being “suboptimal”. Stochastic resonance has been observed, quantified, and described in a plethora of physical and biological systems, including neurons. Being a topic of widespread multidisciplinary interest, the definition of stochastic resonance has evolved significantly over the last decade or so, leading to a number of debates, misunderstandings, and controversies. Perhaps the most important debate is whether the brain has evolved to utilize random noise in vivo, as part of the “neural code”. Surprisingly, this debate has been for the most part ignored by neuroscientists, despite much indirect evidence of a positive role for noise in the brain. We explore some of the reasons for this and argue why it would be more surprising if the brain did not exploit randomness provided by noise—via stochastic resonance or otherwise—than if it did. We also challenge neuroscientists and biologists, both computational and experimental, to embrace a very broad definition of stochastic resonance in terms of signal-processing “noise benefits”, and to devise experiments aimed at verifying that random variability can play a functional role in the brain, nervous system, or other areas of biology.

## Introduction

*Noise* is an all-encompassing term that usually describes undesirable disturbances or fluctuations. In biology, “noise” typically refers to variability in measured data when identical experiments are repeated, or when biosignals cannot be measured without background fluctuations distorting the desired measurement.

Noise is also the fundamental enemy for communications engineers, whose goal is to ensure messages can be transmitted error-free and efficiently from one place to another, at the fastest possible rate. When *random noise* in the form of electronic fluctuations or electromagnetic interference corrupts transmitted messages, this places limits on the rate at which error-free communication can be achieved. If everything else is ideal, then noise is the enemy.

But what if not everything is ideal? Can an ideal system always be implemented in practice? The answer is of course *no*; engineering is about designing systems with tradeoffs between different conflicting objectives. The same could be said of evolution. Given this, there are circumstances—see below—where unavoidably present noise or unpredictable fluctuations can be used purposely, or deliberately introduced to lead to a benefit.

*Stochastic Resonance* (SR) is the name for a phenomenon that is a flagship example of this idea. It has mostly been studied by physicists—for more than 25 years—but may also be familiar to some biologists, as well as to those in many other disciplines. Research into SR has had a colorful evolutionary journey, and extracting important principles and results from the literature can be confusing.

In particular, the paradoxical notion of “good noise” is a double-edged sword for SR researchers. To some, working to understand paradoxes and counterintuitive ideas is a significant curiosity-driven challenge. This has drawn many scientists and engineers to study SR, leading to many interesting and useful theoretical and experimental published results. Others naturally focus only on the ingrained principle of great utility in engineering, where noise needs to be eradicated, and, the more it is present, the more diminished is performance. This preconception can be a sufficient reason for many scientists to ignore or dismiss SR.

The purpose of this essay is to discuss issues that have sometimes clouded the topic. Our first aim is to bring some clarity to the debate and to illustrate the pitfalls and controversies for biologists unfamiliar with stochastic resonance, or who have held only a peripheral interest in the area.

The second aim is to advocate to readers that when they come across studies of SR, they should focus less on the counterintuitive idea of “good noise”, and instead understand SR in terms of “randomness that makes a nonlinearity less detrimental to a signal”. Such a change of focus away from “noise” and on to “helpful randomness” may shift the balance away from skepticism of the form “how can *noise* be good?” toward thinking “does this variability have a useful function?”

Provoking a discussion on this topic is especially timely, given recent increasing interest in the topic of neuronal variability. For example, SR is mentioned in several recent PLoS articles [1]–[4], while a symposium on “Neuronal Variability and Its Functional Significance” was held in conjunction with the 2008 Society for Neuroscience meeting. Furthermore, a recent review on noise in the nervous system [5] has highlighted the multiple sources of neuronal noise and recently developed methods for quantifying them. It is sensibly pointed out in [5] that the nervous system is likely to have evolved methods for both “countering the detrimental effects of noise,” as well as discussing its potential benefits [5].

However, discussion on SR in [5] focuses on a traditional definition that is restrictive in its scope, where the input signal is periodic, necessarily weak compared to the noise, and nearly always “subthreshold”. This special case where noise allows a weak signal to be “detected” often places a focus on single neurons. The concept of beneficial noise by no means needs to be constrained to such conditions, and we discuss in this essay how the definition of SR has evolved beyond the scope discussed by [5].

Example discussions of the many possible ways in which randomness may be useful in biology and neuroscience appear in [6]–[8]. One particularly important idea is that neuronal variability can provide a “probabilistic population code” [9], which allows the brain to represent probability distributions, and perform Bayesian inference [10]. Noise can lead to highly complex phenomena, and other very different benefits beyond signal processing [11],[12]. See also [13] for a review of the physics of biological fluctuations at the molecular level and [14] regarding stochastic gene expression and its advantages. Many of these “noise benefits” could be described as SR—provided that input and output signals can be defined—simply by showing how performance varies as a function of noise level. When this is the case, techniques employed by engineers and physicists to quantify and understand SR may provide theoretical insights in the biological case.

Of course, one can have too much of a good thing: in no way do we suggest that all variability is a sign of exploited randomness. It is quite likely that variability in measurements has no significance, and needs to be filtered or otherwise minimized. But we propound that there are sound reasons why the idea encapsulated by SR should be openly considered. The lesson from studies of SR is that observations of random noise or background fluctuations may be evidence of a source of biological randomness that could potentially be exploited for a functional benefit, whether SR or some other effect. Alternatively, measurements of an information-bearing signal with unpredictable variability may be evidence that randomness has already been utilized to assist representation of information.

We also wish to highlight that unfortunately SR does not always have a good reputation. There are many reasons for this, some of which are touched on in this essay. One reason is that, in the early days of SR research, there was a tendency for perhaps too much enthusiasm, leading to overstatement and exaggeration of what might be achieved by exploiting the idea in applications, and the possibility that it could help us understand brain function. For example, the idea that noise can help “detect weak signals” has sometimes been misstated as proof that our brains may be affected by distant electrical power lines. Even more unfortunately, SR has sometimes been invoked in pseudoscientific contexts, such as explaining why “Native Americans can hear the voices of their ancestors in the noise of the grass”!

While such events remain scarce exceptions to a large body of high-quality scientific research, anecdotal evidence suggests it is timely to discuss why the idea of “noise benefits” should be taken seriously by biologists, whether labeled as SR or not.

The remainder of this essay provides a brief overview of SR research, before progressing to discuss some of the debates and controversies, including those regarding the *definition* of SR. We then outline some thoughts on the future of SR-related research in biology and its potential application in biomedical engineering. We end with six recommendations for biologists to consider when thinking about SR or the possible functional role of neuronal variability. Parts of the following discussion follow along similar lines to argumentation contained in [15].

## A Brief History of Stochastic Resonance

*Stochastic Resonance* (SR), although a term originally used in a very specific context, is now broadly applied to describe any phenomenon where the presence of noise in a nonlinear system is better for output signal quality than its absence. There are several key terms in the previous sentence that require clarification. The first key term is *nonlinear*. Noise cannot be beneficial in a linear system, and it is only the more complex interactions between nonlinearities and randomness that can (sometimes) lead to SR. Another key term is *better*. A wide variety of *performance* measures have been used to quantify *better*, and in nearly all cases it is understood to mean that some aspect of the processing or transmission of a signal is improved. The third key term is the word *noise* itself. Noise is usually associated with words such as *nuisance*, *undesirable*, or *irritating*, and the concept of it being *useful* is apparently contradictory.

This idea can be distilled into stating that whenever SR occurs, it must be true that
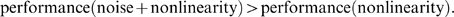



**Figure 1:**
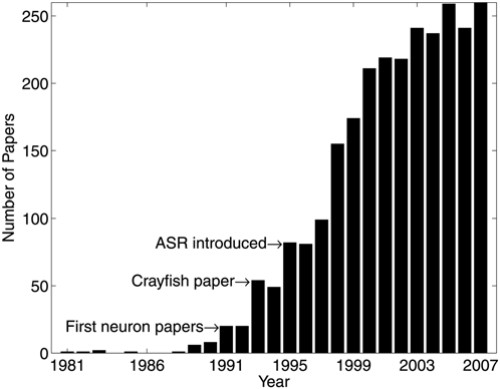
Frequency of stochastic resonance papers by year—between 1981 and 2007—according to the ISI database. There are several epochs in which large increases in the frequency of SR papers occurred. The first of these is between 1989 and 1992, when the most significant events were the first papers examining SR in neural models [47],[48],[118]. The second epoch is between about 1993 and 1996, when the most significant events were the observation of SR in physiological experiments on neurons [49]–[51], the popularization of array-enhanced SR [110], and of Aperiodic Stochastic Resonance (ASR) [107]. Around 1997, a steady increase in SR papers occurred, as investigations of SR in neurons and ASR became widespread.

The term *stochastic resonance* was first used in the context of noise-enhanced signal processing in 1980 by Roberto Benzi, at the 1980 NATO International School of Climatology, as a name for the mechanism suggested to be behind the periodic behavior of the earth's ice ages [16],^17^. The same idea was independently proposed in [18],[19]. *Stochastic Resonance* has been used—according to the ISI Web of Science database—in more than 2,300 publications—see Figure 1. About 20% of papers on SR also contain a reference in the title, abstract, or keywords to the words *neuron* or *neural*, which illustrates the significant interest in studying a positive role for randomness in neural function.

**Figure 2:**
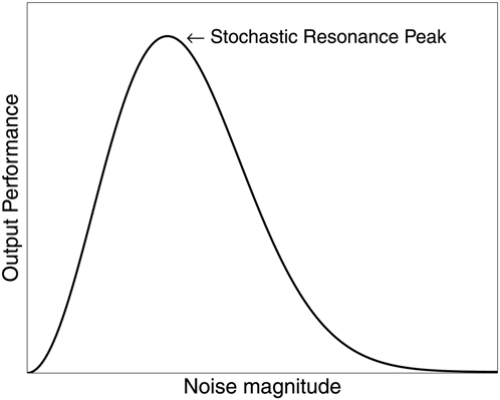
Typical curve of output performance versus input noise magnitude, for systems capable of stochastic resonance. For small and large noise, the performance metric (e.g., SNR, mutual information, Fisher information, correlation, discrimination index) is very small, while some intermediate nonzero noise level provides optimal performance.

The word *resonance* in the term *stochastic resonance* was originally used because the signature feature of SR is that a plot of a performance measure—such as output signal-to-noise ratio (SNR)—against input noise “intensity” has a single maximum at a nonzero value. Such a plot, as shown in Figure 2, has a similar appearance to frequency-dependent systems that have a maximum SNR, or output response, for some *resonant frequency*. However, in the case of SR, the resonance is “noise-induced” rather than at a particular frequency.

Stochastic resonance has been the subject of many thorough technical reviews, some focussing on physical and mathematical descriptions of SR [20]–[28], others on observations of SR in electronic systems and potential applications of SR [15], [29]–[32], and a third group devoted to SR in biology [15], [33]–[35]. For detailed information on theoretical aspects of SR, the reader is referred to these articles. Descriptions of the topic for wider audiences have also appeared [36]–[40].

Stochastic resonance has been widely observed throughout nature—it has been reported and quantified in such diverse systems as climate models [17], electronic circuits [41],[42], differential equations [43],[44], lasers [45],[46], neural models [47],[48], physiological neural populations [49]–[51] and networks [52], chemical reactions [53], ion channels [54], SQUIDs (superconducting quantum interference devices) [55], the behavior of feeding paddlefish [56]–[58], ecological models [59], cell biology [60],[61], financial models [62], psychophysics [63]–[66], carbon-nanotube transistors [67],[68], nanomechanical oscillators [69],[70], organic semiconductor chemistry [71], and even social systems [72].

In its early years, SR was defined only in the very specific context of a bistable system acting on a combination of a *periodic* input signal and random noise. Its definition later evolved to broader contexts, enabling the explosion of interest in the late 1990s—see Figure 1. Interestingly, while SR has been observed in many other systems, its explanatory power for the periodicity of ice ages is still a subject of debate.

### Questions Concerning Stochastic Resonance

There are a number of misconceptions and controversies about stochastic resonance that are apparent in the literature. The following list of questions encapsulates the main points of contention: (i) What is the definition of stochastic resonance?; (ii) Is stochastic resonance exploited by the nervous system and brain as part of the neural code?; (iii) Does stochastic resonance only occur if a signal's power is weak compared to the power of the noise in a system?; (iv) Can stochastic resonance lead to a signal-to-noise ratio gain, and is this consistent with information theory?; (v) Was stochastic resonance known about prior to the first use of the term “stochastic resonance” in 1980?; (vi) How is stochastic resonance different from a signal-processing technique called *dithering*?

Although question (ii) is quite clearly the most interesting scientific question, and seemingly the motivation behind much SR research, the literature reveals that the other questions in the above list have sometimes provided a diversion. The problem is that reaching a consensus on the answers to questions (ii)–(vi) really depends on an agreed-upon answer to question (i).

The broadest possible definition of stochastic resonance is that it occurs when randomness has a positive role in a signal-processing context. Given this definition, *we believe* that the answers to these questions are (ii) yes, although it is difficult to prove, the brain would almost certainly not function as it does if it operated completely deterministically; (iii) no, randomness can have a positive role even if it is only a small amount of randomness; (iv) yes, in the information-theoretic sense, random noise in a system's input signal can lead to a less-noisy output signal, provided that the system is nonlinear and suboptimal; (v) yes, randomness has been known to have a positive role in many circumstances for decades, if not centuries; and (vi) stochastic resonance occurs when dithering is used—dithering can be described as the exploitation of SR [15].

On the other hand, if the definition of stochastic resonance is restricted to its original narrow context, then the answers to questions (ii)–(vi) change to: (ii) maybe—this is yet to be conclusively answered [35], (iii) yes, (iv) no, (v) no, and (vi) dithering is quite different from SR [73].

This discussion is intended to illustrate that the debate on the topics listed above can depend crucially on what one means by *stochastic resonance*.

### Defining Stochastic Resonance

Stochastic resonance is often described as a counterintuitive phenomenon. This is largely due to its historical background, since in the first decade and a half since the coining of the term in 1980, virtually all research into SR considered only systems where the input was a combination of a periodic single-frequency input signal and broadband noise. In such systems, a natural measure of system performance is the output signal-to-noise ratio (SNR), or, more precisely, often the ratio of the output power-spectral-density at the input frequency, to the output noise-floor power-spectral-density measured with the signal present. The noise floor is measured with the signal present, rather than absent, as the output noise may change if the signal is not present. This is because the signal and output noise are generally not additive in a nonlinear system, or, in other words, the output noise is signal-dependent.

For linear signal-processing systems, it is well-known that output SNR is maximized in the absence of noise. This means that observations of the presence of noise in a system providing the maximum output SNR are often seen to be highly counterintuitive—see [39] (p. 149) for further discussion. When it is noted that there are many examples of systems or algorithms where randomness is of benefit, SR does not seem quite so counterintuitive. Examples include: a) Brownian ratchets [74]—mechanical applications of this idea include self-winding (batteryless) wristwatches [75]; b) dithering in signal processing and analog-to-digital conversion [76]–[78]; c) coherence resonance [79],[80]; d) Parrondo's games—the random combination of losing games to produce a winning game [81]; e) random links between clusters of nodes in networks to enhance “small world network” effects [82]; f) noise-induced linearization [83],[84], noise-induced stabilization [85], noise-induced synchronization [86], and noise-induced order [87]; g) the use of mixed (probabilistic) optimal strategies in game theory [88]; h) random switching to control electromagnetic compatibility performance [89]; i) random search optimization techniques, including genetic algorithms [90] and simulated annealing [91]; j) random noise radars—that is, radars that transmit random-noise waveforms in order to provide immunity from jamming, detection, and interference [92]. Further discussion and other examples appear in [15], [39], [93]–[95].

Note that while noise or variability, whether in biology or in engineered systems, may not be truly random—it can, for example, be constant or deterministic (even chaotic)—it is often possible to characterize observations by modeling it as random. Consequently, SR research has tended to focus on the stochastic case. The most common assumption is that the noise is white—that is, constant in power across all frequencies—and Gaussian-distributed. In most cases, changing the distribution or power spectrum of the noise does not change the fact that SR occurs. In this essay, fine details about the noise process are not significant; the important point is that unpredictable variability or fluctuations can be said to be present.

While SR was initially considered to be restricted to the case of periodic input signals, the literature reveals that it now is widely used as an all-encompassing term, whether the input signal is a periodic sine wave, a periodic broadband signal, or aperiodic. An appropriate measure of output performance depends on the task at hand, and the form of input signal. For example, for periodic signals and broadband noise, SNR is often used [24]. When the signal is random and aperiodic, SR can be observed by calculating the mutual information [96],[97] or correlation [98] between the input and output signals, as a function of noise intensity. Another often-used measure is Fisher information [57],[99], which is useful when the goal is to estimate an input signal (or its parameters) from an observed output signal.

These alterations to the original definition have sometimes led to confusion, so we now explicitly discuss the two competing definitions of SR: the conventional definition, and what we contend is the contemporary and more useful definition.

#### Conventional SR: A “bona fide” resonance

The definition of SR, and the word *resonance* itself, have both been objects of debate. In particular, “resonance” is usually thought of in the sense of a resonant frequency, rather than an optimal noise intensity. For the typical early assumptions of periodic signals and small SNRs, “resonance” was more or less resolved as being appropriate, after a new way—using residence time distributions—of looking at SR found that the effect was a bona fide resonance [100]. The reasoning was that *resonance* means a matching of two characteristic frequencies (or physical time scales), and residence time distribution provided a way of interpreting SR in this way [37],[73].

However, there was also some debate about this [101]–[103], and although this definition satisfactorily characterized SR in a manner that allowed “resonance” to be phenomenologically accurate for dynamical bistable systems energized by periodic input signals, it implied that “stochastic resonance” was no longer appropriate for systems consisting of simple “static threshold” nonlinearities. Many papers on SR use the term *static threshold* to describe the nonlinearity being studied. It is used to differentiate between dynamical systems—such as the bistable potential wells typically used in traditional studies of SR—and nondynamical “static” systems [104]. A system is called static when nonlinear deformation—SR cannot occur in a linear system—of an input signal is not governed by time-evolving differential equations, but by simple rules that produce an output signal based on the instantaneous value of the input signal. There are at least two reasons why this has been debated.

First, noise-enhanced behavior in static threshold nonlinearities also occurs in a signal-processing technique known as dithering. Dithering involves deliberately adding a random—or pseudo-random—signal to another signal, prior to its digitization or quantization [76]. It is most often associated with audio or image processing, where the effect of the added noise signal, called the *dither signal*, is to randomize the error signal introduced by quantization. This randomization, although increasing the total power of the noise at the output, reduces undesirable harmonic distortion effects introduced by quantization. As discussed in [15], we believe that the contemporary definition of SR is such that dithering can be described as a technique that *exploits* SR, and the two terms are not mutually exclusive. See also [105].

Secondly, the initial questions about whether noise-enhanced behavior in static threshold systems should be called stochastic resonance relate to whether a bona fide resonance occurs, since no frequency matching occurs for threshold systems [73]. Although this point is technically reasonable, we take the point of view that such questions of semantic nomenclature are no longer relevant. While the “time-scale matching” definition of SR is a satisfactory way of ensuring that “resonance” truly can be said to occur in a restricted subset of noise-enhanced scenarios, it is incongruous with the fact that widespread interest in “stochastic resonance” comes not from whether the term is semantically accurate, but instead from the notion of beneficial randomness.

Furthermore, the strict definition in [100] excludes a significant amount of the content—which could be described in terms of static nonlinearities—of the single most highly cited nonreview paper on SR [106], which was published in 1989 and helped lead to the first great acceleration in SR research, and the first investigations of SR in neurons.

#### Stochastic resonance as “noise benefits”

The term *stochastic resonance* is now used so frequently in the much wider sense of being the occurrence of any kind of noise-enhanced signal processing, that we believe this common usage has, by “weight of numbers”, led to a redefinition. Indeed, electrical engineer Bart Kosko, who made pioneering developments in fuzzy logic and neural networks, concisely defines SR in his popular science book *Noise* as meaning “noise benefit” [39] (pp. 148–149). Kosko also states the caveat that the noise interferes with a “signal of interest”, and we concur that SR can be defined as a “noise benefit in a signal-processing system”, or alternatively “noise-enhanced signal processing”. Put another way, SR occurs when the output signal from a system provides a better representation of the input signal (or of some useful aspect of it) than it would in the complete absence of noise.

Evidence for the emergence of a wider definition includes the many “flavors” of SR that have been described, e.g., aperiodic SR (ASR) [107],[108], array-enhanced SR (AESR) [109],[110], suprathreshold SR (SSR) [15], [111]–[114], system size SR [115], ghost SR [116], and diversity-induced resonance [117]. Although many authors still define SR only in its original narrow context where a resonance effect can be considered to be bona fide, in line with the evolution of languages, words or phrases often end up with a different meaning from their original roots. All the variations mentioned are extensions beyond the original definition of SR, yet they can be classified as “noise benefits” phenomena (in terms of signal enhancement), and our experience is that a majority of researchers are comfortable using the term SR to describe this broadened and richer scope.

We emphasize here the fact that SR occurs only in the context of *signal* enhancement, as this is the feature that sets it apart from many of the list of randomness-enhanced phenomena above, which could all be described as benefiting in some way from noise, and yet cannot all be defined in terms of an enhanced signal. Furthermore, SR is usually understood to occur in systems where there are both well-defined *input* and *output* signals, and the optimal output signal, according to some measure of quality, occurs for some nonzero level and type of noise. In particular, note that *coherence resonance* is often confused with SR. While similar to SR in that an optimum level of noise leads to a benefit in terms of maximally coherent oscillations [79],[80], there is no concept of an *input* signal being converted to an *output* signal.

## The Future of “Noise Benefits” Research in Biology and Biomedical Engineering

While SR has been observed in an increasingly diverse range of biological research areas, such as ion channels [54], behavior [56],[58], ecological models [59], and cell biology [60],[61], interest has mostly focused on neuroscience. We therefore restrict our remaining discussion to the question of how noise—whether thought of in the context of stochastic resonance, or instead as a source of beneficial randomness—may be demonstrated as having functional utility in the brain.

### Does Stochastic Resonance Occur In Vivo in Biological Neurons and Brain Function?

We begin by giving a brief literature review of the main studies on SR that are often cited as indirect evidence for its biological utility. A recent summary of progress for sensory neurons was published in [35].

The first papers investigating SR in neuron models appeared in 1991 [47],[48],[118], with the broader scientific community being introduced to the topic after [48] was discussed by a widely read news article [119]. Research into SR in neurons accelerated—for example, [96], [120]–[123]—after the 1993 observation of SR in physiological experiments where external signal and noise were applied to crayfish mechanoreceptors [49]. Later experimental studies also demonstrated that SR can occur in neurons in the cercal sensory system of a cricket when noise is applied externally [50], and in the human proprioceptive system [51].

Crucially, none of the above-cited papers have been able to prove that neurons “use” SR in a natural setting—the evidence for neurons exploiting SR is only indirect. It can be convincingly argued that these experiments do not prove that neurons utilize SR in any way, because both the signal and the noise were applied *externally* to sensory receptors and neurons. The fact that SR occurs only demonstrates that these cells are nonlinear dynamical systems for which SR effects occur when signal and noise are both added externally. It remains an open question as to whether neurons make use of *internally* generated noise and SR effects. A direct observation of SR would require an external signal, and measurements of *internal* neuronal noise, in vivo [124]. One possibility is that synaptic background activity is a source of beneficial noise [125]—see [5] for related discussion.

One theme of this essay is that it is not paradoxical that randomness may provide benefits in neurons and the brain. It is hardly surprising that the same idea was noticed prior to the first papers on SR and neurons in 1991, as well as in contexts other than SR. For example, in 1971 the first comprehensive analytical studies of the effects of noise on neuron firing demonstrated that noise “smoothes” the firing response of neurons [126],[127]. Later, [128] discusses noise-induced transitions in neural models, and in particular [83] advocates noise as being an important element in signal modulation by neurons.

Interestingly, a constructive role for noise in the context of *neuronal oscillations* in the brain was reported around the same time as the first observations of SR in neural models [129]. Today there is accelerating interest in establishing the function of neuronal oscillations, and the mechanisms that give rise to them [7],[130],[131]. Various theories have been proposed, and random variability may have an essential role in ensuring the robustness of either synchronized oscillating populations [7],[11] or the emergence of fast oscillations in local field potentials [132]. It may also be the case that variability in the spike-trains of individual sensory neurons is important for ensuring that an overall population produces tuning curves optimized for information transmission [133],[134].

Whether a positive role for random noise in such contexts can be called SR or not depends, even with the broad definition used in this paper, on whether it is useful to define an input and output signal. The big picture, though, is independent of whether it should be called SR or not. Instead, the fact that random noise can provide a benefit is the key idea, regardless of what one calls it. This essay is placed in the context of SR, since it provides a paradigm for understanding why it should not be surprising that random variability can have a function.

### Debates about SR and Detection Theory

We now briefly outline one of the central arguments for dismissing SR, i.e., the conclusion that *optimal* signal detection is incompatible with the fact that SR is observed when detection performance is non-monotonically decreasing with increasing input SNR. This difficulty has been discussed several times in the literature. Of particular relevance to biologists are the opposing viewpoints of Tougaard and Ward et. al. Tougaard's initial critique concluded that “Improving detection by means of stochastic resonance is thus a suboptimal strategy” [135]. Ward et al. rebutted the idea that SR is always such an “epiphenomenon of nonoptimal criterion placement”, using counterexamples beyond the scope of [135], and also a focus on whole organism performance rather than on single neurons [65].

In response [136], Tougaard revisited the problem, and conceded that the models of [65] led to SR due to nonlinearities in the production of neural action-potentials, while detection theory based on the “receiver” part of the neuron still decreased monotonically with increasing noise. Tougaard's conclusion was that “the role of the noise is to compensate for the inherent nonlinear process of spike generation … ” and “The detectability … decreases monotonically with input noise level, in full correspondence with the central dogma of signal detection theory”.

Largely overshadowed—although touched upon—by the focus of this debate, is the fact that “optimal detectors” may not be efficiently implemented, and that optimal detection may not have been the primary driving factor of evolution. Furthermore, the focus of Tougaard is on single detectors, rather than on populations of them. This is also a limitation in a similar discussion of the fact that SR is a suboptimal strategy [137]. The conclusion that SR in threshold systems is simply a way to overcome the incorrect threshold setting seems to have led many to think that making use of noise is a suboptimal means of designing a system. The contrasting viewpoint is that noise is ubiquitous; since it is virtually impossible to remove all noise completely from systems, design methods should consider the effects of SR, and that various design parameters, such as a threshold value, may in some circumstances need to be set in ways that make use of the inherent noise to obtain an optimal response.

One example of this is suprathreshold stochastic resonance (SSR) [15], [111]–[114]. In this variant of SR, which relies on a parallel population of “sensors” or neurons collectively encoding a common stimulus—see, e.g., [138] for illustration—noise benefits do disappear if the overall population is optimized [139]. However, the optimal solution is extremely complex and not plausibly achievable by real neurons. A much simpler and robust solution is to maintain a configuration that while suboptimal and exhibiting noise benefits is close enough to optimal over a large range of input SNRs. Furthermore, SSR is a form of aperiodic stochastic resonance, meaning that detection is not at stake, but instead the goal could be information transmission, signal classification, or signal compression [140].

So, while Tougaard's analysis is an important reminder that SR “… is not, as is sometimes misunderstood, able to make otherwise nondetectable signals detectable”—see also [141]—it seems clear from this debate that while detection theory might suggest that increasing noise can never improve detection, this in no way implies that noise cannot have a useful role, due to (i) the complexities of combining detection with nonlinear information encoding; (ii) the possibility that the theoretically “optimal” detector is by no means optimal in a broad sense; and (iii) that *detection* is not necessarily the signal-processing goal. See [142]–[144] for some related discussion.

Indeed, very recently electronic engineers have started exploring the possibility of improving necessarily suboptimal detectors by randomizing them [145]–[148], and of deliberately employing noise when device limitations do not allow any other kind of detector optimization [68],[149].

### Biomedical Applications of SR

A different form of indirect evidence for SR existing naturally in biology is successful biomedical applications. A particularly notable example is the use of electrically generated subthreshold stimuli in biomedical prosthetics to improve human balance control and somatosensation [150]–[156]. This work led to James J. Collins winning a prestigious MacArthur Fellowship in October 2003 [154].

Another proposed application inspired by SR, as first suggested by Morse and Evans in 1996 [157], is that of enhanced cochlear implant stimulation strategies. Cochlear implants can restore hearing to the profoundly deaf by direct electronic stimulation of the auditory nerve using a surgically implanted electrode array [158]. Numerous authors have since advocated the exploitation of SR in this area, e.g., [159]–[162].

The basic idea is that several sources of unpredictable variability present in fibers of the auditory nerve during normal hearing [163],[164] are known to be absent in deafened ears. It is hypothesized that a well-controlled random component in the output of cochlear implant electrical signals would therefore stimulate nerve fibers in a more natural way that may lead to improved hearing. It has been proposed that healthy hearing exploits SSR as a way of enabling the afferent nerve fibres that synapse with inner hair cells to encode more information about sound waveforms than would be possible in the absence of randomness [15],[160]. The prospect of reintroducing natural variability to more closely mimic the natural activity of healthy nerve fibres is taken very seriously, with at least two independent approaches to practical implementation [165]–[167].

The same principle of making the output of biomedical prosthetics more like biology has also been applied in mechanical life-support ventilators. In order to more closely replicate natural breathing, random noise was introduced into the operation of the artificial ventilator, and it was found that it enhanced performance in several ways [168]. Later modeling supported this result, and interpreted it as a form of SR [169]. See [170] for a review.

The current acceleration of research into medical bionics such as brain–machine interfaces, and electronic sensory prosthetics (e.g., cochlear implants, auditory brainstem implants, and retinal implants for restoring vision), means there is an increasing need to understand how unpredictable fluctuations may be exploited in biology. Such understanding may be critical for the successful design of some types of future bionics.

### Concluding Remarks: Six Recommendations for Biologists

From an engineer's perspective, if it can be established that SR plays an important role in the encoding and processing of information in the brain, and that it somehow provides part of the brain's superior performance to computers and artificial intelligence in some areas, then using this knowledge in engineering systems may revolutionize the way we design computers, sensors, and communications systems.

For biological science, rather than view SR as a specifically defined phenomenon of limited scope, we advocate thinking about SR in terms of the broad idea of “noise benefits”, and as a reminder that ideal systems often cannot be engineered in practice. When this is the case, it is necessary to make the best of a suboptimal situation, such as exploiting noise to advantage. This principle holds for evolution as well. If there are nonlinearities involved, then it is easy to imagine that organisms evolved to make the best possible use of noise and fluctuations that are unavoidably present.

With this in mind, we present six recommendations for biologists to consider when performing literature searches on stochastic resonance or noise in biology, and when trying to understand whether biological noise may have some useful functional role.

1. It is not necessary, and indeed often meaningless, to define performance in terms of signal-to-noise ratio (SNR) [171],[172]. Likewise, it is not necessary to focus on *detection*. Output SNR is unlikely to be a useful way of quantifying SR in biology, since it is a measure designed for linear systems and artificial electronic systems. If instead the goal is to prove that biological function may rely on random noise, it makes more sense to measure variations in function with changing internal or input noise level, in whatever manner would normally be the case.
2. Adding noise to external stimuli cannot prove that neurons or brain function depend on consistently available internal sources of randomness, i.e., on *endogenous neural noise* [124]. The challenge is to devise an experiment that can remove naturally occurring healthy variability and demonstrate that function is impaired solely due to that removal. This has been termed *intrinsic stochastic resonance*—see [124] for discussion. Alternatively, it may be possible to empirically verify that function or performance is impaired due to the loss of a source of variability through disease or accident.
3. It is not necessary to focus on periodic input signals. For example, for experiments on sensory neurons, it would be most useful to utilize stimuli based on “natural scenes” [173],[174] that mimic the inputs expected to be processed by those neurons, and to assess how noise might benefit coding of such signals.
4. It is not necessary to focus on subthreshold signals. Noise benefits can occur for suprathreshold signals, in particular if a common signal is processed by a population of neurons [111]. Further, a “hard-threshold” is by no means a necessary condition for SR—see [175] and references therein.
5. If noise benefits are found, explaining why they occur will likely be in terms of constraints that mean an alternative, superior, non-noisy mechanism is not efficiently feasible or robust [96].
6. Describing SR as a *technique* is misleading. This is because it implies that SR is a signal-processing strategy in its own right, and confuses cause with effect. Instead, by recalling that observations of frequency resonance in oscillators are analogous to SR, we suggest SR can instead be accurately referred to as an *observed phenomenon* that can potentially be exploited—e.g., “stochastic resonance was observed to occur when the level of random noise was changed, with peak performance occurring when the noise variance was *x*”. In this circumstance, the system itself is capable of SR, and the *technique* that is employed is that of modifying the noise intensity.
